# Supplementary material for: Microhaplotype deep sequencing assays to capture Plasmodium vivax infection lineages
Source: Nat Commun. 2025 Aug 5;16:7192. doi: 10.1038/s41467-025-62357-x (PMC12325997; doi:10.1038/s41467-025-62357-x)
Supplement: Supplementary file 1 — Supplementary Information [file 41467_2025_62357_MOESM1_ESM.pdf]

Supplementary Information for:

Microhaplotype deep sequencing assays to capture *Plasmodium vivax* infection lineages

Mariana Kleinecke[1*], Edwin Sutanto[2*], Angela Rumaseb[1], Kian Soon Hoon[1], Hidayat Trimarsanto[1,2], Ashley Osborne[1], Paulo Manrique[3,4], Trent Peters[5], David Hawkes[5], Ernest Diez Benavente[6], Georgia Whitton[7], Sasha V Siegel[7], Richard D Pearson[7], Roberto Amato[7], Anjana Rai[1], Nguyen Thanh Thuy Nhien[8], Nguyen Hoang Chau[8], Ashenafi Assefa[9], Tamiru S Degaga[10], Dagimawie Tadesse Abate[10], Awab Ghulam Rahim[11], Ayodhia Pitaloka Pasaribu[12], Inge Sutanto[13], Mohammad Shafiul Alam[14], Zuleima Pava[1], Tatiana Lopera-Mesa[15], Diego Echeverry[16], Tim William[1,17], Nicholas M Anstey[1], Matthew J Grigg[1], Nicholas P Day[18,19], Nicholas J White[18,19], Dominic P Kwiatkowski[7**], Aimee R Taylor[20], Rintis Noviyanti[21], Daniel Neafsey[3,4], Ric N Price[1,18,19], Sarah Auburn[1,18] ***

1. Menzies School of Health Research and Charles Darwin University, Darwin, Australia
2. Exeins Health Initiative, South Jakarta, Indonesia
3. Harvard T.H. Chan School of Public Health, Boston, Massachusetts, USA
4. Broad Institute, Boston, Massachusetts, USA
5. Australian Genome Research Facility, Brisbane, Australia
6. Laboratory of Experimental Cardiology, Department of Cardiology, University Medical Center Utrecht, Utrecht, the Netherlands
7. Wellcome Sanger Institute, Hinxton, UK
8. Oxford University Clinical Research Unit, Hospital for Tropical Diseases, Ho Chi Minh City, Vietnam
9. Ethiopian Public Health Institute, Addis Ababa, Ethiopia
10. College of Medicine & Health Sciences, Arba Minch University, Arba Minch, Ethiopia
11. Afghan International Islamic University, Kabul, Afghanistan
12. Universitas Sumatera Utara, Medan, Indonesia
13. Faculty of Medicine, University of Indonesia, Jakarta, Indonesia
14. Infectious Diseases Division, International Centre for Diarrhoeal Disease Research, Bangladesh (icddr,b), Dhaka, Bangladesh
15. Universidad de Antioquia, Medellin, Colombia
16. Departamento de Microbiología, Facultad de Salud, Universidad del Valle, Cali, Colombia
17. Queen Elizabeth Hospital, Kota Kinabalu, Malaysia
18. Centre for Tropical Medicine and Global Health, Nuffield Department of Medicine, University

of Oxford, Oxford, United Kingdom

19. Mahidol-Oxford Tropical Medicine Research Unit, Mahidol University, Bangkok, Thailand
20. Institut Pasteur, University de Paris, Infectious Disease Epidemiology and Analytics Unit, Paris, France
21. Eijkman Molecular Biology Research Center, National Research and Innovation Agency, Cibinong, Indonesia

*These authors made equal contributions. **Deceased. ***Corresponding author: Sarah.Auburn@menzies.edu.au

Contents

Supplementary Figure 1. Genomic locations of the 97 nuclear genome markers. 3

Supplementary Figure 2. Overview flowchart of Plasmodium spp sample genotyping and analysis. ... 4

Supplementary Figure 3. Specificity of the assays. 5

Supplementary Figure 4. Assay sensitivity using serial dilutions. 8

Supplementary Figure 5. Pvmtcox1 qPCR Ct and parasite density against genotyping yield. 9

Supplementary Table 1. Assay sensitivity in the P. vivax serial dilutions. 10

Supplementary Figure 6. Genotyping success by parasite density in dried blood spot samples. 11

Supplementary Table 2. Concordance in SNP-based genotype calling between amplicon sequencing and whole genome sequencing (WGS) data at 10% threshold 12

Supplementary Table 3. Concordance in SNP-based genotype calling between amplicon sequencing and whole genome sequencing (WGS) data at 1% threshold 13

Supplementary Figure 7. Allele depth differences between WGS and amplicon sequencing data. 14

Supplementary Figure 8. Microhaplotype-based within-host diversity trends at the Provincial level. 16

Supplementary Figure 10. IBD-based spatial patterns using microhaplotype data at IBD thresholds from 6%. 19

Supplementary Table 4. Plasmodium spp. classification. 20

Supplementary Table 5. Prevalence of orthologous drug resistance markers in each country. 22

Supplementary Note 1. *P. vivax* rhAmpSeq Library Preparation 23

Supplementary Note 2. Bioinformatic Pipeline for rhAmpSeq data analysis 29

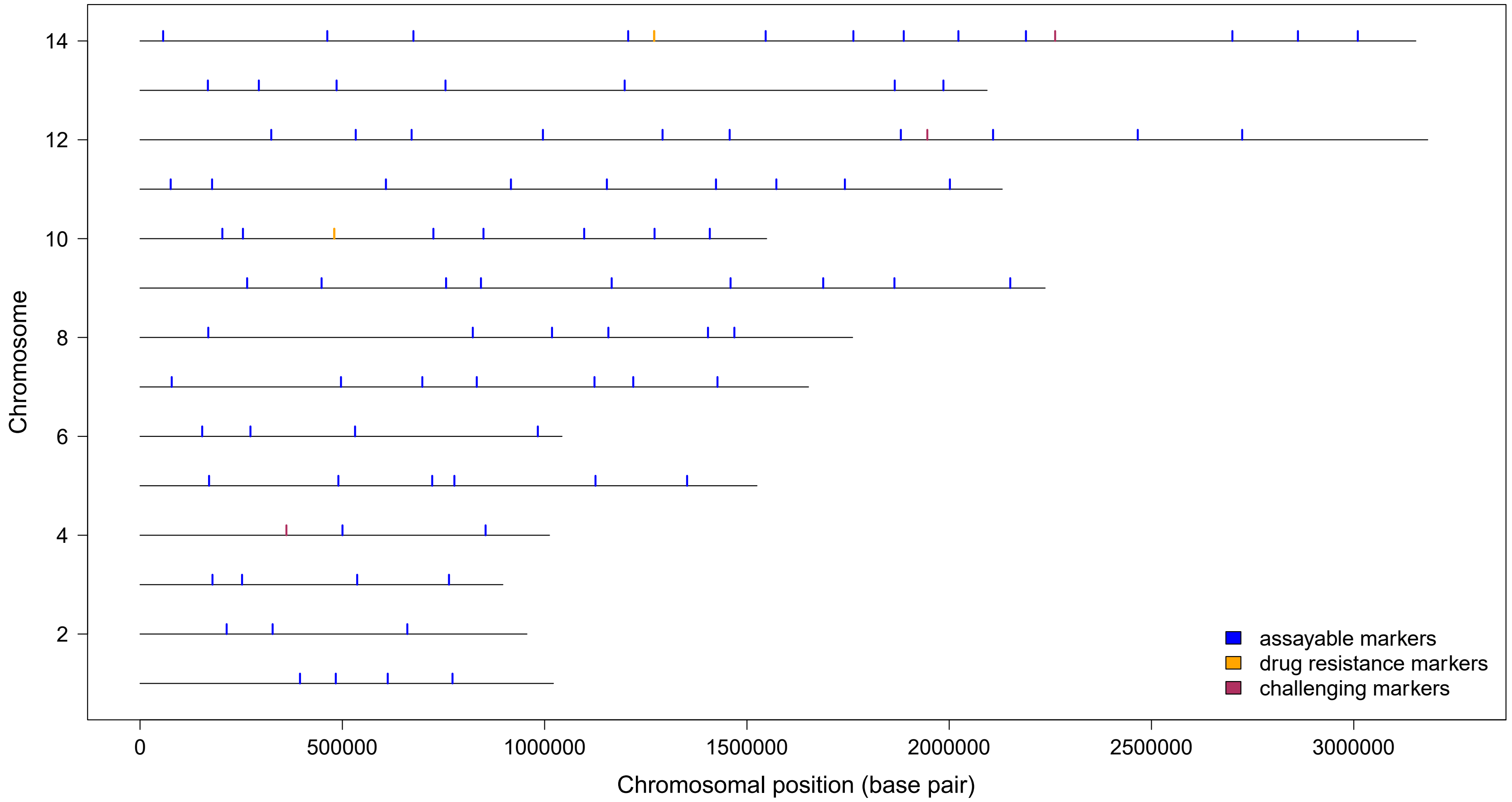

Supplementary Figure 1. Genomic locations of the 97 nuclear genome markers. Note, excluding the mitochondrial locus. Markers described as "challenging" displayed low read-pair depth with *dada2* output; markers 64721 (Chromosome 4), 354590 (Chromosome 12) and 466426 (Chromosome 14) (see Figure 2b).

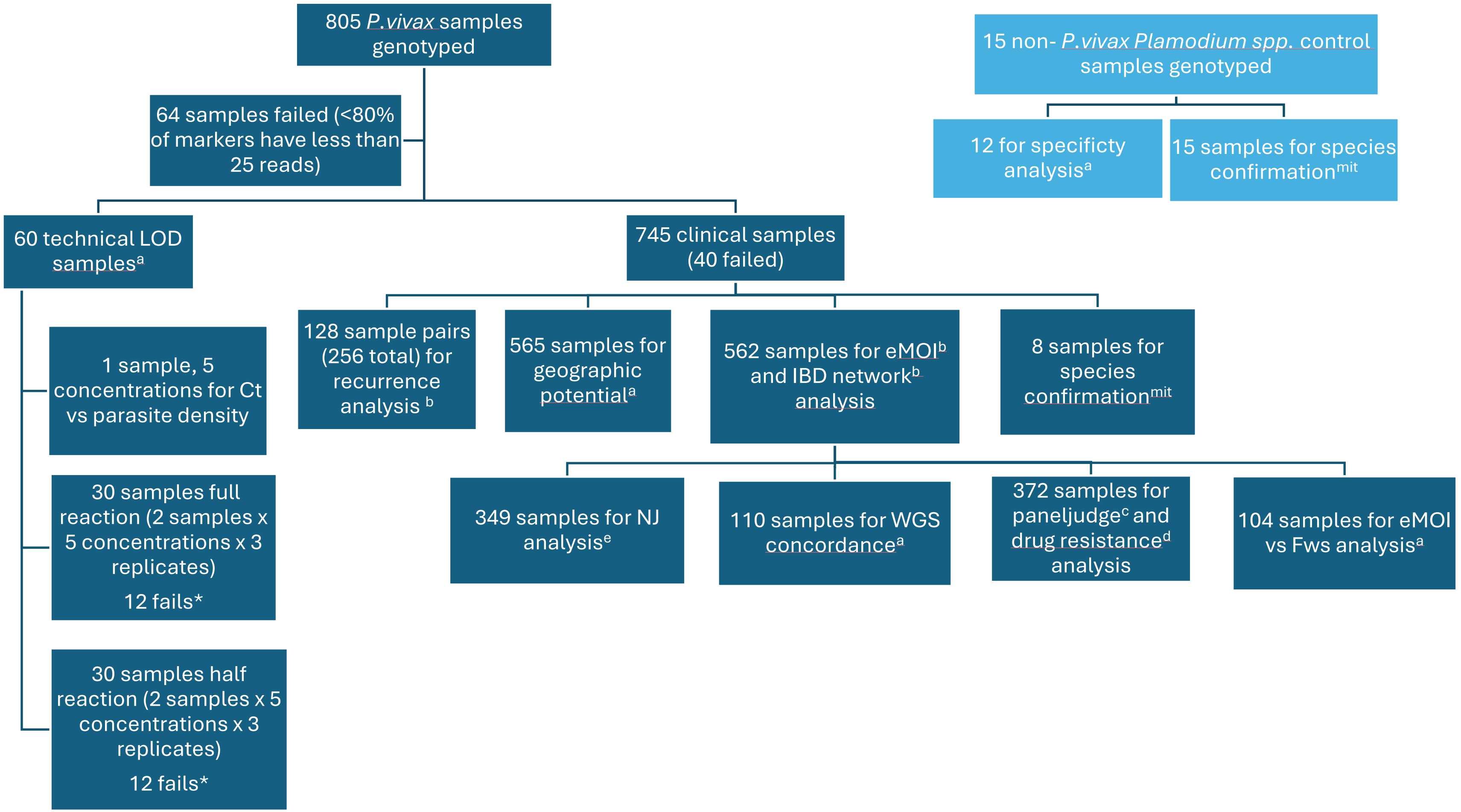

Supplementary Figure 2. Overview flowchart of *Plasmodium* spp sample genotyping and analysis. *P. vivax* samples are presented in dark blue boxes and other species are presented in light blue boxes. * Samples failed here were the 2 lowest concentrations (all under 9.6 ng/ul) for all three replicates. [a]97 markers used (all except mitochondria). [b] 93 markers used (excluding mitochondria and 4 drug resistance candidate markers). [c] 91 markers used (excluding mitochondria, Mhap markers 354590 and 419038, and 4 drug resistance candidate markers). [d] Only markers in *pvmdr1* and *pvdhps*. [mit] only the mitochondrial region. [e] 73 Mhap markers used, and amplicon sequencing data combined with pre-existing WGS data on 379 low complexity samples. The 20 markers excluded from NJ

analysis are 15441, 44595, 50287, 54827, 64721, 67489, 100236, 151535, 178560, 185264, 212462, 267505, 274564, 304503, 344819, 354590, 389038, 416631, 419038 and 466426.

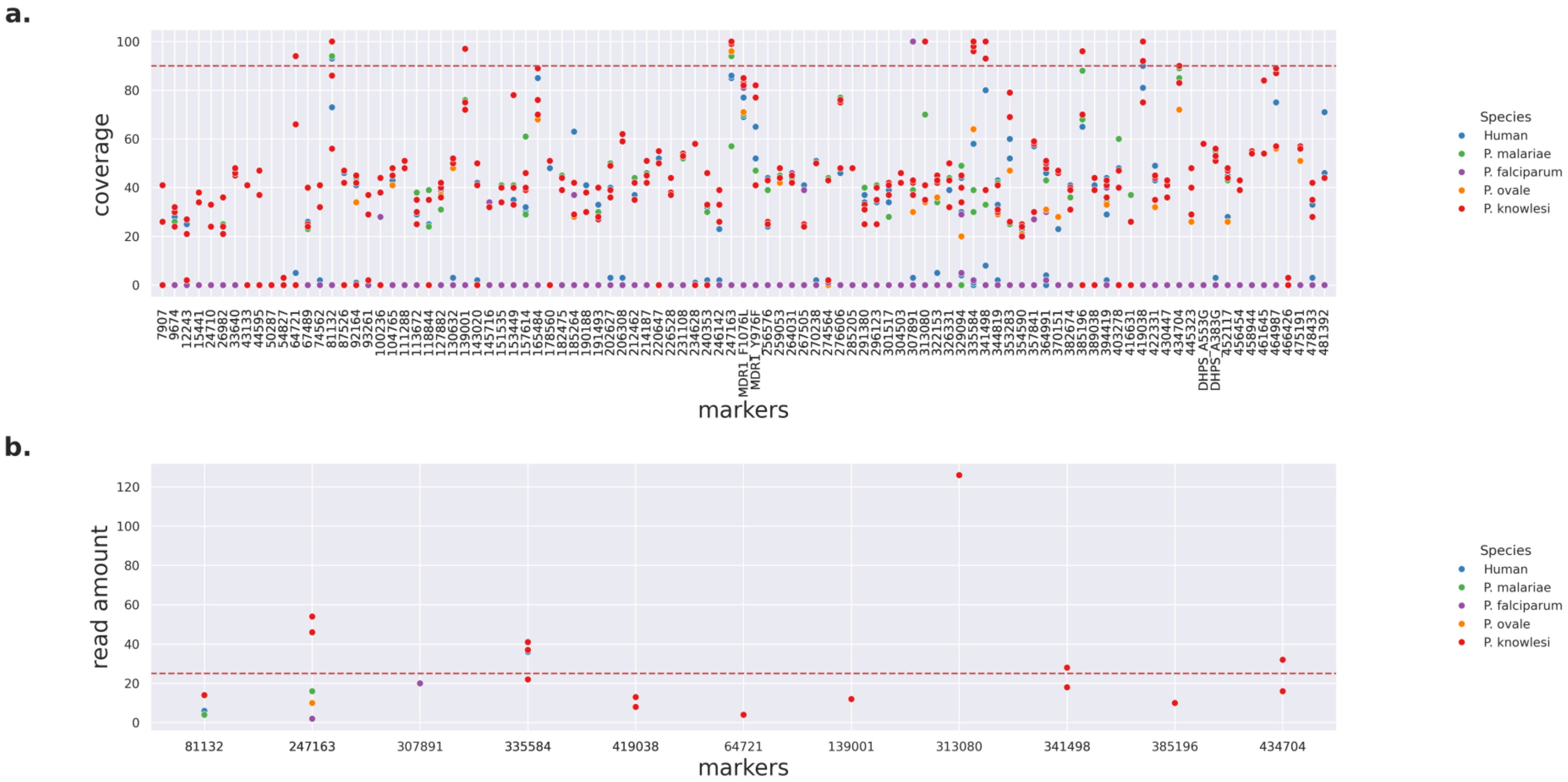

Supplementary Figure 3. Specificity of the assays. Panel a) presents coverage over all markers aside from the mitochondrial *Plasmodium* spp. marker in non-*P. vivax* samples. Genotypes with coverage over 0.9 (90%; dotted line) were investigated for read amount as per panel b). At a cut-off of 25 (dotted line), aside from 7 *P. knowlesi* genotypes, non-specific calls were removed. A threshold of 25 was set to reduce background noise in downstream analyses, although it should be noted that different thresholds may be required for different data sets. The results presented here comprise 96-plex and 384-plex runs as majority of

the negative controls were processed on a pilot 96-plex run; in theory, this enhances the potential to capture non-target amplicons. Data is presented on 12 independent non-vivax samples including 4 human, 2 *P. falciparum*, 2 *P. malariae*, 1 *P. ovale* and 3 *P. knowlesi* mono-species samples.

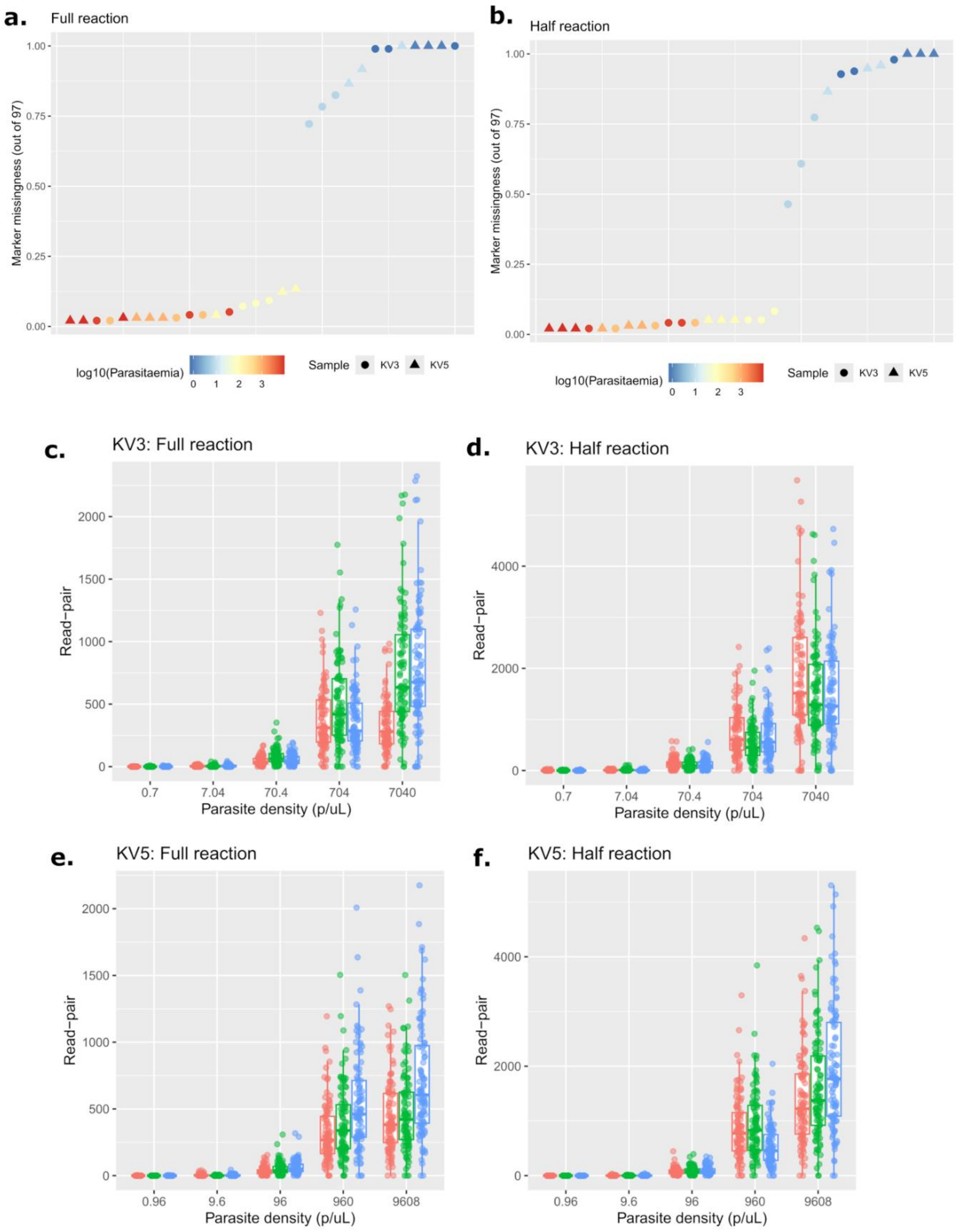
a.
Full reaction
Marker missingness (out of 97)
1.00
0.75
0.50
0.25
0.00
log10(Parasitaemia)
0 1 2 3
Sample
KV3
KV5
b.
Half reaction
Marker missingness (out of 97)
1.00
0.75
0.50
0.25
0.00
log10(Parasitaemia)
0 1 2 3
Sample
KV3
KV5
c.
KV3: Full reaction
Read-pair
2000
1500
1000
500
0
0.7
7.04
70.4
704
7040
Parasite density (p/uL)
d.
KV3: Half reaction
Read-pair
4000
2000
0
0.7
7.04
70.4
704
7040
Parasite density (p/uL)
e.
KV5: Full reaction
Read-pair
2000
1500
1000
500
0
0.96
9.6
96
960
9608
Parasite density (p/uL)
f.
KV5: Half reaction
Read-pair
4000
2000
0
0.96
9.6
96
960
9608
Parasite density (p/uL)

86

Supplementary Figure 4. Assay sensitivity using serial dilutions. Panels a) and b) illustrate the number of fails across the 97 markers (excluding the mitochondrial marker) in two independent *P. vivax* serial dilutions, samples KV3 and KV5, each with triplicate reactions (coloured pink, green and blue). Panel a) presents the data for full reactions (20 ul reaction mix in library preparation PCR step 1, comprising 11 ul DNA). Panel b) presents data for half reactions (10 ul reaction mix in PCR step 1, comprising 5.5 ul DNA) reactions. At parasite densities at and above 70-96 parasites per ul, marker missingness was less than 15 (less than the arbitrary 20% sample fail threshold applied here) in both the full and half reaction experiments. Panels c) to f) illustrate the distribution of read counts in the same serial dilution experiments Each boxplot presents the median, minimum and maximum read count. The read counts for the half reactions were generally higher than those for the full reactions. This result was unexpected and may reflect modest differences in the amount of each library contributed to the final pool for the run, as the half and full reactions were pooled separately. Also of note, within each full and half reaction, the KV3 sample, with estimated 7,040 p/ul starting density, had slightly higher sensitivity than the KV5 sample, which has estimated 9,600 p/ul starting density. This trend was also unexpected and may reflect inaccuracies in the estimation of the amount of DNA in each sample as microscopy measures do not account for differences in DNA abundance between different *P. vivax* life cycle stages. Microscopy estimates also do not account for free DNA (i.e. DNA outside of the cells) or potential DNA degradation. All results reflect *P. vivax* sensitivity on 384-plex runs on a MiSeq instrument.

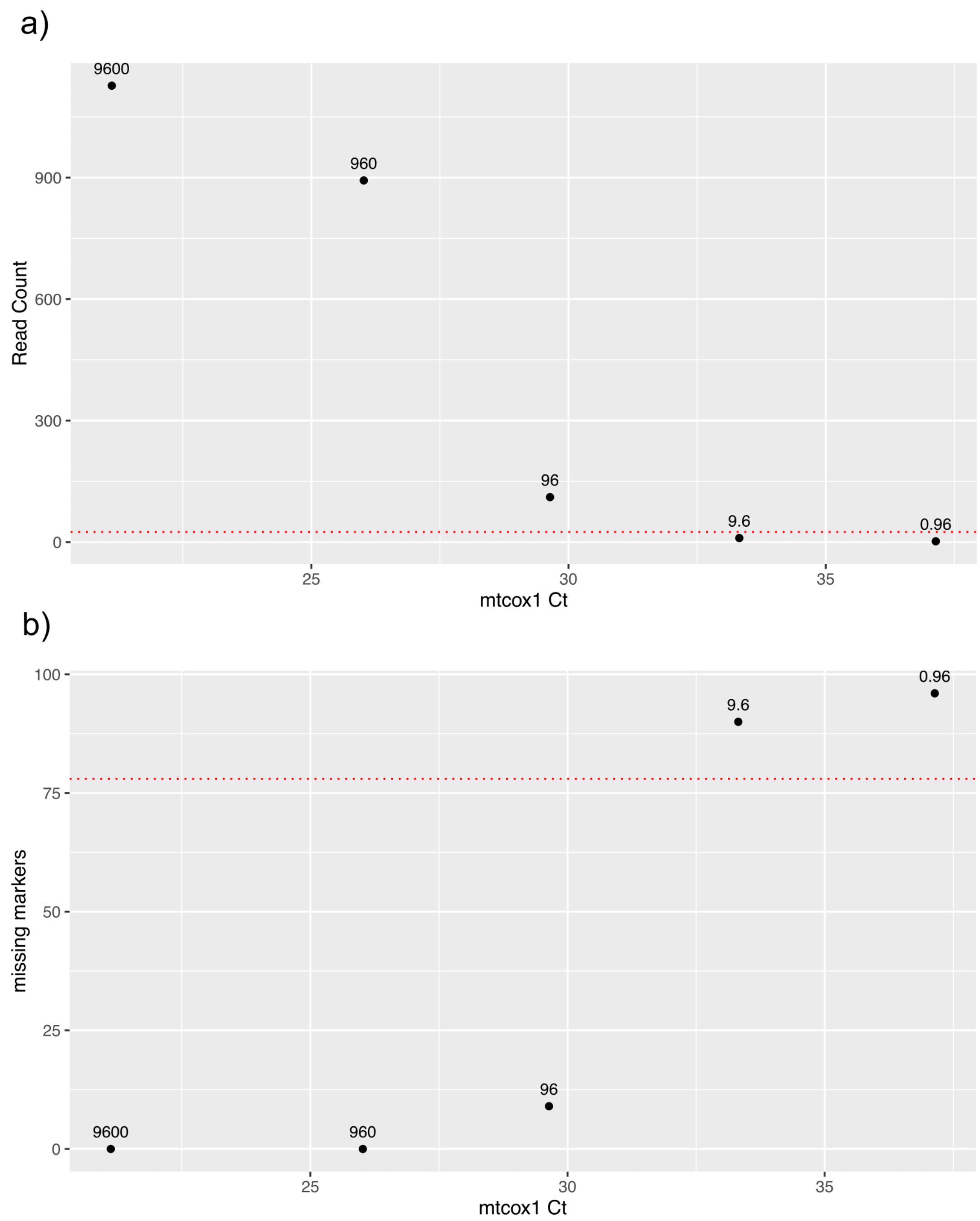

Supplementary Figure 5. Pvmtcox1 qPCR Ct and parasite density against genotyping yield. Data is presented on a serial dilution from one independent sample (KV5), with parasite densities labeled above each point. The *pvmtcox1* Ct scores reflect an average across triplicates. Panel a) presents the average read count, with the dashed red line delineating the threshold for an average of 25 reads over all markers (minus mitochondrial marker), which is reached at and above 96 parasites per microliter blood. Panel b) presents the percentage of missing markers, with the dashed red line delineating the threshold for 80% failed markers, which the 96 parasites per microliter and higher parasite densities sit well below.

**Sample**	**Parasite density (p/ul)**	**Average rhAmpSeq read count[a]**	**Average *pvmtcox1* Ct[b]**	***delta* Ct**	***Amount of markers missing[a]***
KV3	7040	1303	N/A	N/A	1.3
KV3	704	862	N/A	N/A	1.7
**KV3**	**70.4**	**129**	N/A	N/A	6.3
KV3	7.04	15	N/A	N/A	79.3
KV3	0.7	4	N/A	N/A	95.7
KV5	9,600	1127	21.12	4.90	0
KV5	960	893	26.02	3.62	0
**KV5**	**96**	**111**	**29.64**	**3.68**	**9**
KV5	9.6	10	33.32	3.82	90.3
KV5	0.96	2	37.14	N/A	96

Supplementary Table 1. Assay sensitivity in the *P. vivax* serial dilutions. Summary of the read counts derived from the rhAmpSeq assay and cycle threshold (Ct) in the *pvmtcox1* PCR in serial dilutions of two independent *P. vivax* samples, KV3 and KV5. The KV3 and KV5 dilutions above which a minimum of 78 markers (80%) are present with a yield of >= 25 reads on average in the rhAmpSeq assay are highlighted in bold. [a] Averaged across replicates (n=3) across 97 markers in the assay (excluding the mitochondrial marker which has multiple copies per cell). [b] Averaged across triplicates.

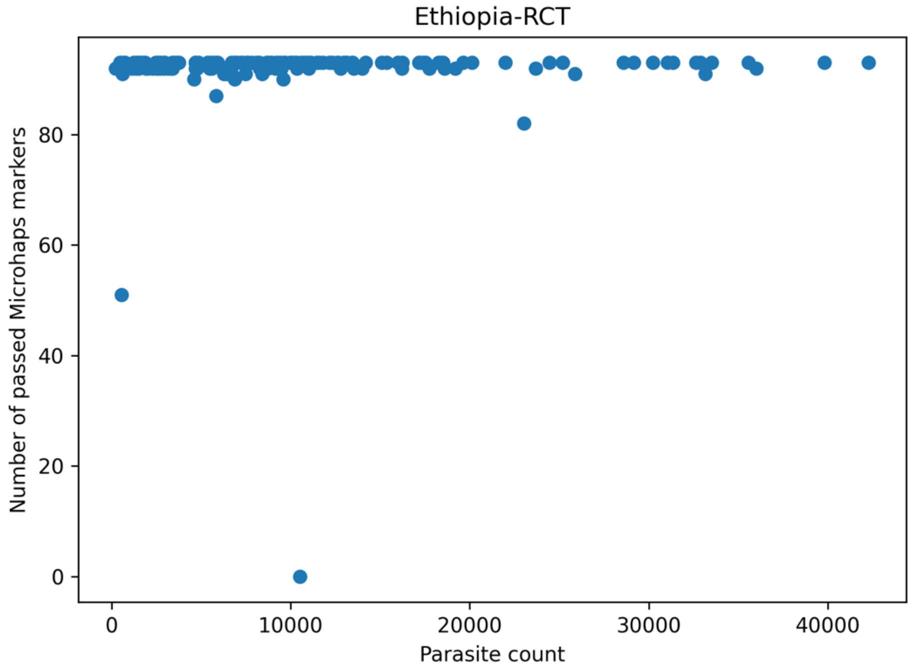

Supplementary Figure 6. Genotyping success by parasite density in dried blood spot samples. The number of passed microhaplotype markers is shown against parasite count (parasites per ul blood) in order of increasing density. Only two samples had less than 79 (80%) marker fails; one representing a low parasite count and one a high parasite count (>10,000 parasites per ul blood). Data is presented on 148 independent samples.

**10% minor allele threshold**	Mhap homozygous reference	Mhap homozygous alternate	Mhap heterozygous	Mhap genotype fail
WGS homozygous reference	54.14% (24,031/44,386)	0.13% (58/44,386)	0.09% (39/44,386)	1,092
WGS homozygous alternate	0.20% (90/44,386)	39.88% (17,703/44,386)	0.42% (188[a]/44,386)	
WGS heterozygous	2.55% (1,133/44,386)	0.16% (73/44,386)	2.41% (1,070/44,386)	
WGS genotype fail	1,235			37

Supplementary Table 2. Concordance in SNP-based genotype calling between amplicon sequencing and whole genome sequencing (WGS) data at 10% threshold. The data is derived from genotype calls derived from the VCF pipeline at 425 biallelic SNPs in 110 independent *P. vivax* samples with high quality WGS and amplicon sequencing data using the default 10% minor allele (with a minimum minor allele depth of 2) threshold. For this analysis, we arbitrarily imposed a stricter minimum depth of 25 for a genotype to be called from the amplicon sequencing data, while the WGS were called with a minimum depth of 5 as is standard for *P. vivax*. The numerator and denominator reflect the number of genotypes meeting the given criteria and the total number of successful genotyping calls across the dataset (44,386) respectively. [a]Ten genotype respectively with a second alternate allele.

<table>
<tr><th>1% minor allele threshold</th><th>Mhap homozygous reference</th><th>Mhap homozygous alternate</th><th>Mhap heterozygous</th><th>Mhap genotype fail</th></tr>
<tr><td>WGS homozygous reference</td><td>54.16% (24,040/44,386)</td><td>0.08% (37/44,386)</td><td>0.1% (41/44,386)</td><td rowspan="3">1,092</td></tr>
<tr><td>WGS homozygous alternate</td><td>0.20% (90/44,386)</td><td>38.83% (7,235/44,386)</td><td>1.48% (656[a]/44,386)</td></tr>
<tr><td>WGS heterozygous</td><td>2.58% (1,147/44,386)</td><td>0.07% (33/44,386)</td><td>2.47% (1,096/44,386)</td></tr>
<tr><td>WGS genotype fail</td><td colspan="3">1,235</td><td>37</td></tr>
</table>

Supplementary Table 3. Concordance in SNP-based genotype calling between amplicon sequencing and whole genome sequencing (WGS) data at 1% threshold. The data is derived from genotype calls derived from the VCF pipeline at 425 biallelic SNPs in 110 independent *P. vivax* samples with high quality WGS and amplicon sequencing data using a 1% minor allele threshold (with a minimum minor allele depth of 10). The same stricter minimum depth of 25 was imposed on amplicon sequencing data while a minimum depth of 5 was required for WGS data. The numerator and denominator reflect the number of genotypes meeting the given criteria and the total number of successful genotyping calls across the dataset (44,386) respectively. [a] 12 genotypes with a second alternate allele.

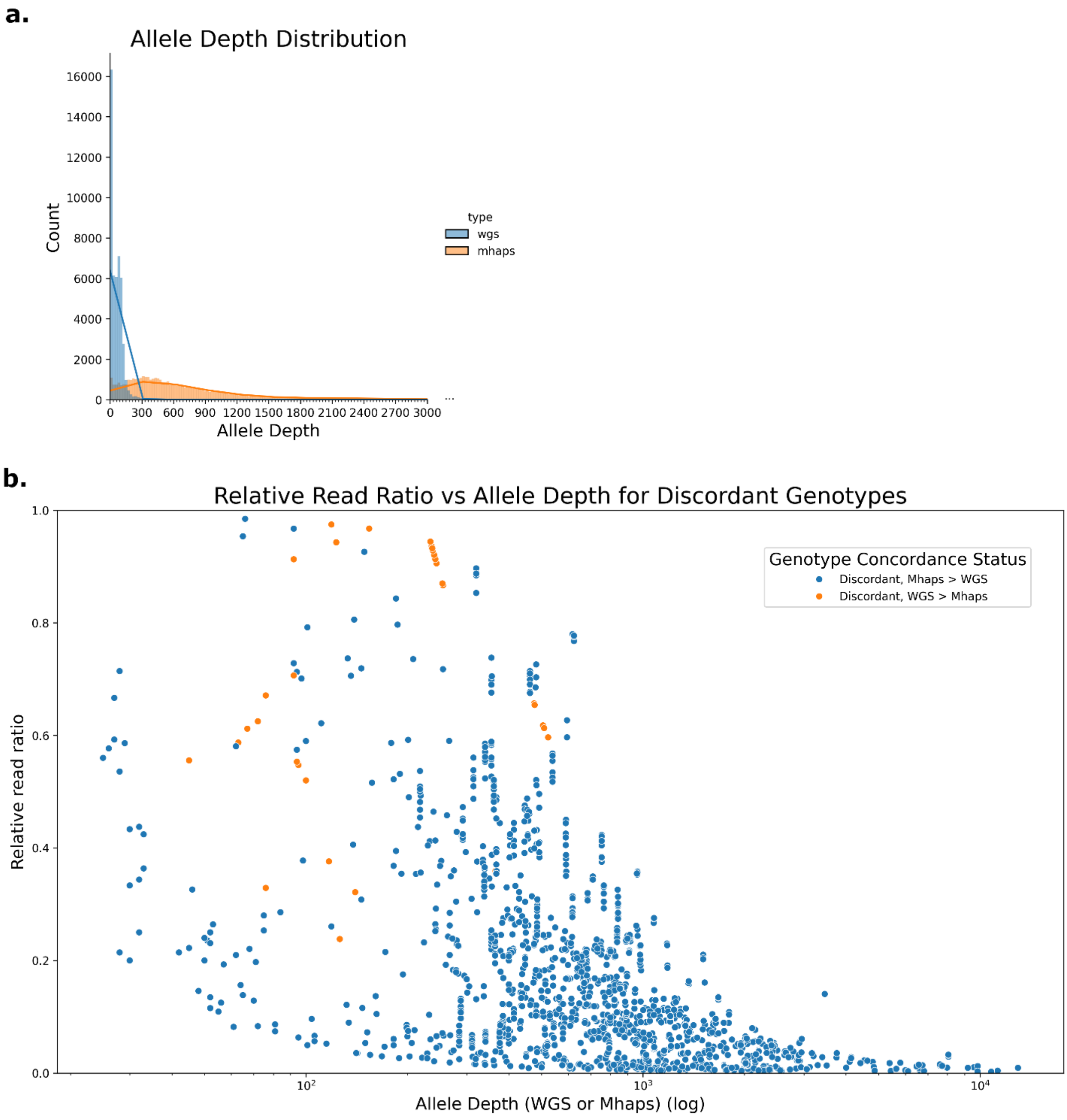

Supplementary Figure 7. Allele depth differences between WGS and amplicon sequencing data. Panel a) presents the distribution of allele depths (x-axis) for genotypes (genotype count on y-axis) derived from whole genome sequencing (WGS) versus amplicon sequencing (mhaps). The genotypes derived from amplicon sequencing exhibit a higher allele depth than those derived from WGS. Panel b) provides a scatterplot illustrating the relationship between allele depth and the relative read ratio for genotypes that showed discordance between WGS and amplicon sequencing (Mhaps). Each point in the plot represents a single discordant genotype. The x-axis displays the allele depth (on a logarithmic scale) for the given genotype, using the depth from either WGS or Mhaps. The y-axis shows the relative read ratio calculated as the ratio of the lower allele depth against the higher allele depth,

with a relative read ratio of 1 indicating equal allele depth between the two sequencing methods. Discordant genotypes are colored based on which method had higher allele depth: blue points indicate cases where Mhaps had a higher allele depth, while orange points represent cases where WGS had a higher allele depth. The plot highlights that a large proportion of discordant genotypes occur at high allele depths, with a clear trend of Mhaps exhibiting significantly higher read depth than WGS at these sites.

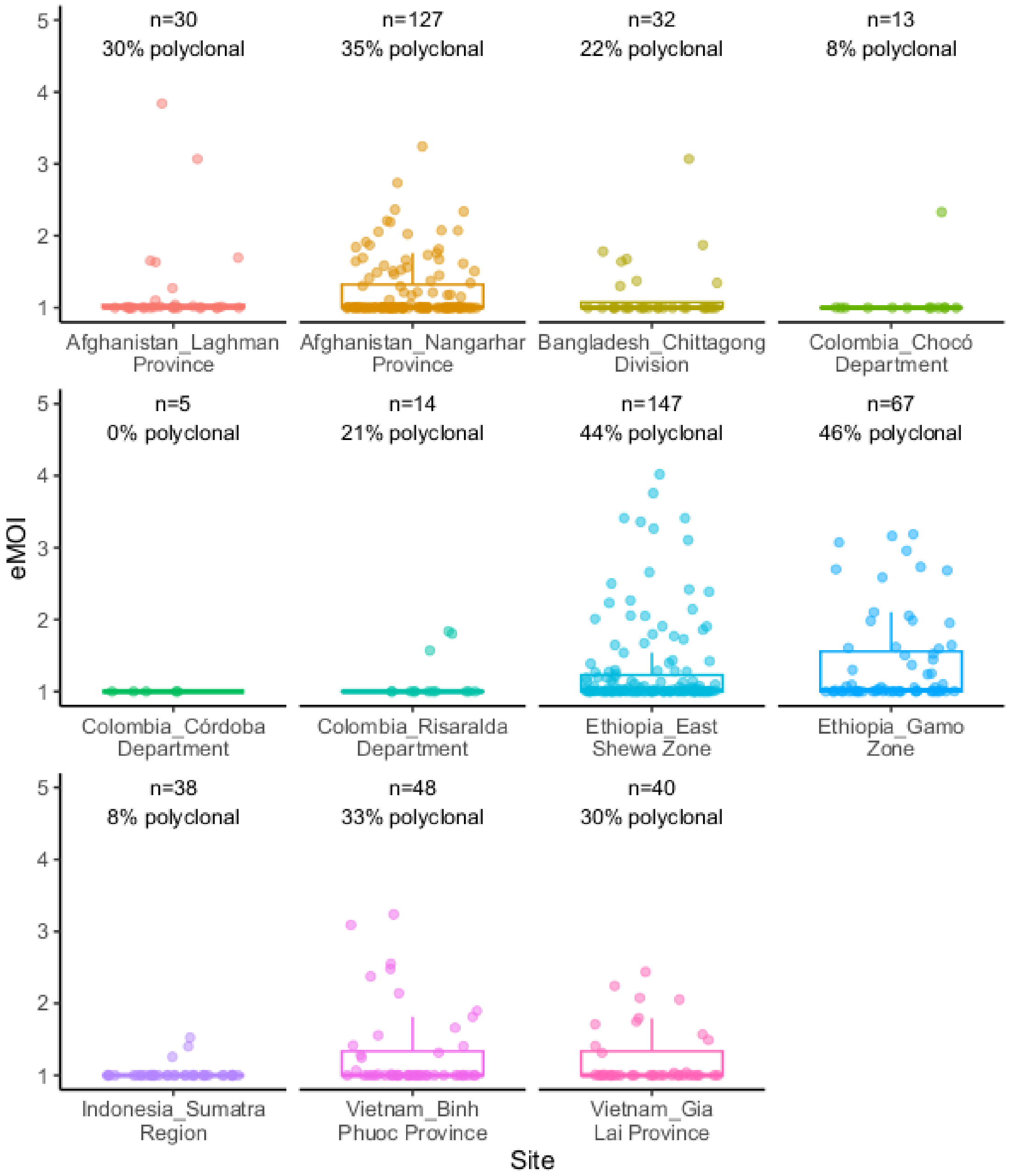

Supplementary Figure 8. Microhaplotype-based within-host diversity trends at the Provincial level. The boxplots present the median, interquartile range and min and max eMOI for the given provinces. Overall, high concordance is observed between the two datasets. Data is presented on a total of 562 independent (non-replicate and non-recurrent) cases are presented, with sample sizes for each province indicated above each distribution. Each site is presented in a different colour.

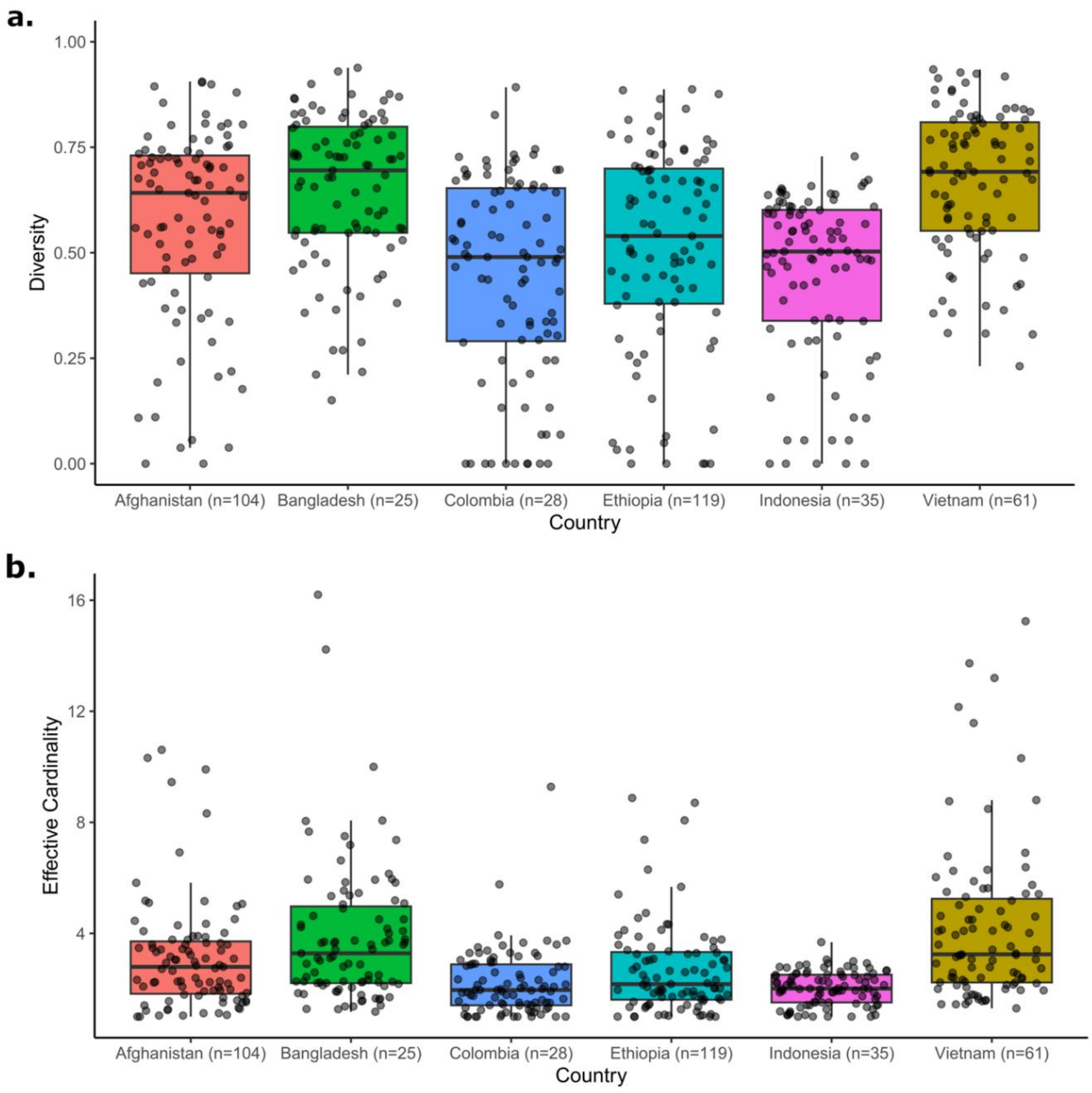

Supplementary Figure 9. Marker diversity by country. Panel a) presents heterozygosity measures and panel b) presents effective cardinality scores in n=372 independent samples. Each boxplot presents the median, interquartile range and min and max value for the heterozygosity (panel a) and effective cardinality (panel b). Each country is presented in a different colour.

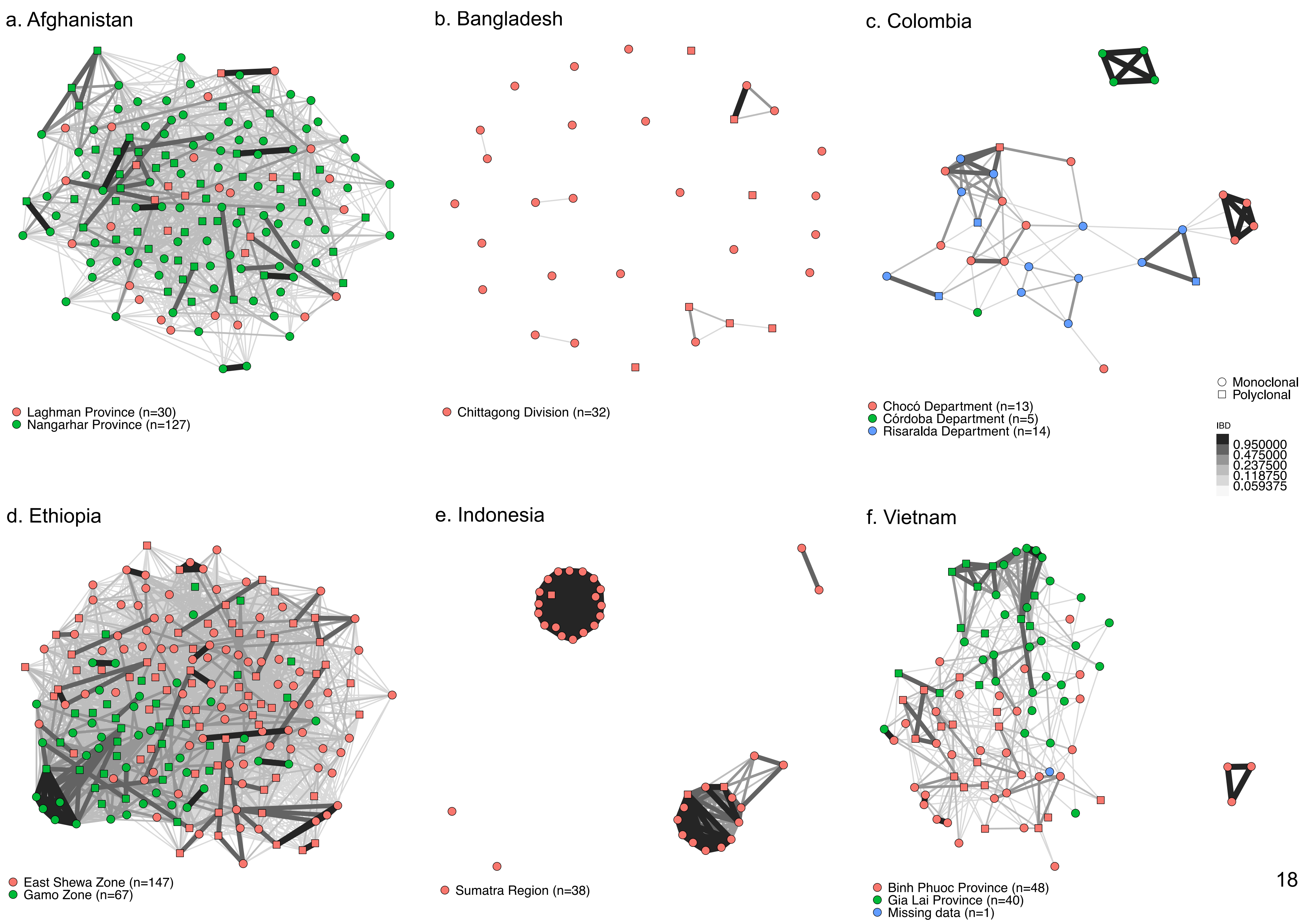
a. Afghanistan
Laghman Province (n=30)
Nangarhar Province (n=127)
b. Bangladesh
Chittagong Division (n=32)
c. Colombia
Chocó Department (n=13)
Córdoba Department (n=5)
Risaralda Department (n=14)
Monoclonal
Polyclonal
IBD
0.950000
0.475000
0.237500
0.118750
0.059375
d. Ethiopia
East Shewa Zone (n=147)
Gamo Zone (n=67)
e. Indonesia
Sumatra Region (n=38)
f. Vietnam
Binh Phuoc Province (n=48)
Gia Lai Province (n=40)
Missing data (n=1)

Supplementary Figure 10. IBD-based spatial patterns using microhaplotype data at IBD thresholds from 6%. Panels a) to f) present networks illustrating IBD-based connectivity between infections in Afghanistan (a), Bangladesh (b), Colombia (c), Ethiopia (d), Indonesia (e) and Vietnam (f). Each shape reflects an infection, colour-coded by site, and with shapes reflecting monoclonal (circle) versus polyclonal (square) infections. For each country, connectivity (illustrated by connecting lines on a grey scale between shapes) is presented at IBD thresholds ranging from ≥0.06 (thin, very light grey lines) to ≥0.95 (thick, black lines). The baseline sample positions are based on the ~6% (0.059) IBD output. IBD measures were calculated on the microhaplotype calls using *DCifer* software. All plots were generated using data on independent infections with sample sizes shown within the plots.

Sample	Run	PCR-based Species	Country	Read Count	Coverage	Mitochondrial *Plasmodium* spp
QECK_1_NV22	Run 2	*Homo sapiens*	N/A	2	0.450	N/A
QECK_2_NV22	Run 2	*Homo sapiens*	N/A	0	0.000	N/A
QECK_3_NV22	Run 3	*Homo sapiens*	N/A	0	0.000	N/A
Hu	Run 11	*Homo sapiens*	N/A	0	0.000	N/A
RDM_47_NV22	Run 2	*P. falciparum*	Indonesia	5,236	1.000	*P. falciparum*
K1	Run 11	*P. falciparum*	Unknown	49,276	1.000	*P. falciparum*
RDM_44_NV22	Run 2	*P. malariae*	Uganda	52	0.990	*P. malariae*
RDM_45_NV22	Run 2	*P. malariae*	Uganda	26	0.980	*P. malariae*
RDM_67_NV22	Run 2	*P. ovale*	Uganda	150	0.980	*P. ovale*
KK_64_NV22	Run 2	*P. knowlesi*	Malaysia	2398	1.000	*P. knowlesi*
KK_107_NV22	Run 2	*P. knowlesi*	Malaysia	74	0.990	*P. knowlesi*
KK_95_NV22	Run 2	*P. knowlesi*	Malaysia	66	0.990	*P. knowlesi*
AF001_099_D0	Run 3	*P. vivax*	Afghanistan	1384	1.000	*P. vivax*
M004	Run 3	*P. vivax*	Bangladesh	158	0.990	*P. vivax*
05027V-1	Run 2	*P. vivax*	Colombia	896	0.990	*P. vivax*
CQE001	Run 3	*P. vivax*	Ethiopia	1058	1.000	*P. vivax*
ID004020_D0	Run 6	*P. vivax*	Indonesia	160	0.990	*P. vivax*
MV1	Run 2	*P. vivax*	Malaysia	1050	1.000	*P. vivax*
MIDAS-129	Run 2	*P. vivax*	Sudan	2158	1.000	*P. vivax*
VN001_002	Run 3	*P. vivax*	Vietnam	168	0.990	*P. vivax*
RDM_neg82_pos_CQE_94	Run 8	*P. vivax + P. falciparum*	N/A	118	0.990	*P. vivax*
RDM_neg47_pos_CQE_20	Run 8	*P. vivax + P. falciparum*	N/A	140	0.990	*P. vivax + P. falciparum*
K1_pos_CQE_29	Run 8	*P. vivax + P. falciparum*	N/A	290	1.000	*P. vivax + P. falciparum*

Supplementary Table 4. Plasmodium spp. classification. Details of the *Plasmodium* spp. Classification determined at the mitochondrial amplicon in 4 human controls, 8 independent non-vivax infections, 8 independent *P. vivax* infections, and 3 artificially mixed *P. vivax* and *P. falciparum* infection selected for representation of a range of countries. Low depth and coverage of the mitochondrial amplicon was confirmed in the 4 human controls, and concordance between PCR-based and mitochondrial

classification of *Plasmodium* spp. was confirmed in all malaria infections. The number of samples in each run were as follows: run 2 (n=96), runs 3-6 (n=384), run 7 (n=48), run 8 (n=107) and run 11 (n=48).

Gene	Chr	Position	Mutation	Drug	Freq, % (no./No.) Afghanistan	Freq, % (no./No.) Bangladesh	Freq, % (no./No.) Colombia	Freq, % (no./No.) Ethiopia	Freq, % (no./No.) Sumatra, Indonesia	Freq, % (no./No.) Vietnam
pvmdr1 (PVP01_1010900)	10	479908	F1076L	CQ	100 (104/104)	100 (25/25)	4 (1/28)	100 (119/119)	100 (35/35)	90 (55/61)
pvmdr1 (PVP01_1010900)	10	480207	Y976F	CQ, AQ+SP	0 (0/103)	8 (2/25)	4 (1/28)	45 (53/119)	100 (35/35)	79 (48/61)
pvdhps (PVP01_1429500)	14	1270401	A553G	Antifolate	0 (0/104)	20 (5/25)	0 (0/28)	0 (0/119)	0 (0/35)	3 (2/61)
pvdhps (PVP01_1429500)	14	1270911	A383G	Antifolate	2 (2/104)	24 (6/25)	86 (24/28)	7 (8/119)	51 (18/35)	85 (52/61)

Supplementary Table 5. Prevalence of orthologous drug resistance markers in each country. Mutation prevalence was calculated with homozygous calls only. Abbreviations: Chr, chromosome; Freq, Frequency; AQ, amodiaquine; Chr, chromosome; CQ, chloroquine; MQ, mefloquine; SP, sulfadoxine-pyrimethamine.

Supplementary Note 1. *P. vivax* rhAmpSeq Library Preparation

Individual rhAmpSeq primers (see Appendix I) with standard desalting purification were purchased from Integrated DNA Technologies (IDT) and reconstituted to 100µM with IDTE, pH 7.5. rhAmp PCR reactions were prepared with 4x rhAmpSeq Library Mix 1 (IDT) and varied primer concentrations optimized to achieve moderately uniform depth across amplicons (range 0.2 – 5.7µM). For full reactions, a total of 11µl genomic DNA (regardless of parasite density) was included in a total PCR reaction volume of 20µl. The rhAmp PCR reaction was run using the following settings: Enzyme activation: 10 min at 95°C**;** 14 cycles of Amplification: Denaturation: 15 sec at 95°C; Annealing: 8 min at 61°C**;** Enzyme deactivation: 15 min at 99.5°C. The rhAmp PCR products were then diluted in nuclease-free water using 1 in 20 dilutions to a total volume of 100µl. Indexing PCR reactions were prepared with the following components: 2µl of nuclease-free water, 2µl of xGEN 10nt Unique Dual Index, 5µl of 4x rhAmpSeq Library Mix 2 (IDT), and 11µl of diluted PCR1 product. The Indexing PCR reaction was run using the following settings: Enzyme activation: 3 min at 95°C**;** 24 cycles of Amplification: Denaturation: 15 sec at 95°C; Annealing: 30 sec at 60°C; Extension: 30 sec at 72°C**;** Final Extension: 1 min at 72°C. Half reactions were run using half the quantity of input genomic DNA and rhAmpSeq Library Mix 1 for the rhAmp PCR, and half the rhAmp PCR product input and rhAmpSeq Library Mix 2 for the Indexing PCR reactions. The thermocycling conditions for the half reactions were the same as for the full reactions.

After amplification, we pooled up to 384 samples with 5µl of each individual library combined in a 1.5mL LoBind Microcentrifuge tube. The pooled libraries were purified to remove primer dimers using 0.7X SPRI beads (Beckman Coulter). After incubating for 10 minutes at room temperature, a magnetic rack was used to separate beads and remove the supernatant. Beads were washed twice with freshly prepared 80% ethanol and left to stand for 3 minutes to enable any leftover ethanol to evaporate. The library (of preferred interest size) was then eluted in 22µl of IDTE, pH 8 (IDT). The success of library purification was evaluated by running pre- and post- bead cleanup libraries with capillary electrophoresis using an Agilent 4150 TapeStation system using D1000 reagents and ScreenTape. The pooled libraries were quantified using Collibri™ Library Quantification Kit and diluted accordingly to 4nM concentration. The diluted library was the sequenced on an Illumina MiSeq or miniSeq platform at 9pM final loading concentration with 10% PhiX. Sequencing was conducted using the Illumina v2 kit following the protocol for paired end 150 bp reads.

Appendix I - *Plasmodium vivax* rhAmpSeq Primer Sequences

AssayID_IDT	Chr	Start	End	Target	*Marker type	PrimerSequence_FWD	PrimerSequence_REV
RH.9CAFEBAA3F384A0Z0Z	1	395355	395521	7907	Mhap	/rhSeq-f/ACN CCC CAA ATG TGA ATA ArCT TCC /GT4/	/rhSeq-r/ACG TGG CTA CTA CCC CArG TGG T/GT3/
RH.2960ABE5E62C470Z0Z	1	483735	483883	9674	Mhap	/rhSeq-f/TCC AAA CTN AGC TCC TTG ATrG TTG T/GT3/	/rhSeq-r/CAA CTT TGG CAT CCT CTA TAA CArC GGA T/GT1/
RH.B2905CD19921440Z0Z	1	612225	612348	12243	Mhap	/rhSeq-f/ACC TGG AAA CTC CCT TGT TrGC AAT /GT2/	/rhSeq-r/ATA CGA ATT CGC ATC AGA CGrG AGA G/GT4/
RH.79D714EC610C448Z0Z	1	772093	772211	15441	Mhap	/rhSeq-f/TCC GAA CCA TCG CTG TTA rCCA CT/GT2/	/rhSeq-r/CTG CCC CTT TCT CCA GAG rCAG TA/GT2/
RH.964D47B0B088468Z0Z	2	213958	214133	24710	Mhap	/rhSeq-f/ACC TGG AAT GCT CCA AAA ATT rCCT TG/GT1/	/rhSeq-r/GGT AGT GTA CAG GGA AAT CAC rCCC GA/GT1/
RH.AC2609A2062C4D4Z0Z	2	327552	327739	26982	Mhap	/rhSeq-f/TTC GCA ACA AGA GGA GCA AArC ATA G/GT2/	/rhSeq-r/CCG TCA AAT GGT AAA GCG TrGA AGN /GT4/
RH.C0DE1AD20EB44E6Z0Z	2	660463	660614	33640	Mhap	/rhSeq-f/GCA GCG CAT GGA AAG TAT TGrC TAG A/GT3/	/rhSeq-r/TGA TCC ACT GCC TTT TGG TAG rCAT TC/GT3/
RH.ADD86C5C7CEE4FBZ0Z	3	178937	179094	43133	Mhap	/rhSeq-f/TGG CAT AGC TGC GAA GTT rATT CA/GT3/	/rhSeq-r/NTC CAC GTG GCT GTA TrAG GGG /GT2/
RH.AC6AF27F13E1456Z0Z	3	252061	252170	44595	Mhap	/rhSeq-f/TTC GAT TTG GAA TCC CCT TrCT GCN /GT4/	/rhSeq-r/CAA GAA AAC CCC ACC TTT GrCA CAA /GT1/
RH.7702F5B5FFEA486Z0Z	3	536620	536711	50287	Mhap	/rhSeq-f/TCC CTG CTG AAG GAC TCrC GAG C/GT1/	/rhSeq-r/ACT CAC CGN CAA CGT TrGG GCG /GT3/
RH.10A2E23284CC46CZ0Z	3	763605	763660	54827	Mhap	/rhSeq-f/CTC CTG GCA TGG ACC CrCA CCT /GT3/	/rhSeq-r/TAN AGC AGG CGG TAG AGrC TTC C/GT1/
RH.00A0825CCA024A4Z0Z	4	361787	361899	64721	Mhap	/rhSeq-f/GAC CAA AGA GGA GAA AAC GArA AAA C/GT4/	/rhSeq-r/TCC TCT TTC ACC TGC TCG rCAT GT/GT3/
RH.9A9B6B1CE8ED464Z0Z	4	500160	500273	67489	Mhap	/rhSeq-f/CAT GAG GTA GTA GCT CTT CGA rCGA GT/GT1/	/rhSeq-r/GCT TCC CCA TGG AGG GrCC TCC /GT3/
RH.FC72753AD141415Z0Z	4	853861	853944	74562	Mhap	/rhSeq-f/CTG CNC AGT TTG ATC AGT CrCA CCC /GT1/	/rhSeq-r/CTT CAT TAT TTC GAA TGG CTT TCT rGGA AG/GT1/
RH.FDE3FDC2B0774C8Z0Z	5	170476	170547	81132	Mhap	/rhSeq-f/TGA ATC CTC CGA AAA CGA TTC rCTC AG/GT3/	/rhSeq-r/GCA GTC TGA AGA TTC TGA TGrA AGA A/GT3/
RH.34E5DDB83D7D4F7Z0Z	5	490198	490244	87526	Mhap	/rhSeq-f/CCC TCA TCA ATC ACT TCT TCC TArC AGA A/GT2/	/rhSeq-r/CTT TTG CGC AAA TAA ATC CAA GTrG AAA C/GT4/
RH.34F85DB3B3264F2Z0Z	5	722013	722089	92164	Mhap	/rhSeq-f/GAC GAG CAA ATT TAA GAA GCT CTC rGTA GC/GT1/	/rhSeq-r/CTG CCA CAT CCT AAA TCA CAT ACT TrCA TAA /GT4/
RH.6A1D6D2210DF49BZ0Z	5	776828	776973	93261	Mhap	/rhSeq-f/ATG AGA TTC ACA CTG TAG TCG GrGG CAG /GT2/	/rhSeq-r/CCT CGT ATC GTT CCT TNA GTC rCTC TT/GT4/
RH.83D956F8758C4BBZ0Z	5	1125680	1125748	100236	Mhap	/rhSeq-f/CCA CGC AGA GTG CTT TTrC CAT C/GT1/	/rhSeq-r/CTT GTC TCA CCG CTG CrCC TCA /GT3/

RH.42DF2C222BA74C2Z0Z	5	1352101	1352165	104765	Mhap	/rhSeq-f/GGT TCG ACA TTA TGA GTA GAC ACrG TTT G/GT4/	/rhSeq-r/CGT TGA CCT TTT GGG AAA CAT ArCA CAT /GT1/
RH.46D4266C768744DZ0Z	6	153460	153574	111288	Mhap	/rhSeq-f/CAA TTT TGC GAG GGC TAT TCrC GCA C/GT1/	/rhSeq-r/TCA CAT GAA GTG TGC AGT TrGC TGG /GT2/
RH.5312BCFBF8D8457Z0Z	6	272690	272815	113672	Mhap	/rhSeq-f/TTA GAA GTC AAT GCG ACG CrCA GAT /GT1/	/rhSeq-r/TCT GAA TGA CCT TCC GGA rGCT GG/GT2/
RH.B21F2DF1DA6A419Z0Z	6	531256	531393	118844	Mhap	/rhSeq-f/AGT CCT GCT CTC AGG GrGT CCT /GT4/	/rhSeq-r/AGG ATG CTC ACC AGG CrGG ACA /GT2/
RH.4133F987D8A44E5Z0Z	6	983157	983333	127882	Mhap	/rhSeq-f/GGA TAT GGA AGG CAN CGG ATrA TTC C/GT4/	/rhSeq-r/TAA TCC CTT CCC CAT TCT CGrA ATC C/GT4/
RH.50720784FEED46AZ0Z	7	78143	78172	130632	Mhap	/rhSeq-f/GTT CTT TTA AAT AAT GCA CCT TTT TCG rCCA TC/GT1/	/rhSeq-r/GAA AAC CAA AAT AGA TGA AAG TTT ACA AArC AGT G/GT2/
RH.3E77B333C8DB4D2Z0Z	7	496520	496613	139001	Mhap	/rhSeq-f/AGA ATG TGT CGG ATT TTC GAT TAG rGAC TC/GT1/	/rhSeq-r/ACA ACC GCA TGT ACA ATC TTT TrGA AGG /GT4/
RH.DEF0990049A641CZ0Z	7	697428	697579	143020	Mhap	/rhSeq-f/CTC ACT CAT GGA TGG GTA CAT AGrA AAA C/GT4/	/rhSeq-r/GGG AAC CAC ATT TAC AGA TTA TCA ArAT GAG /GT2/
RH.0AB7872636F3460Z0Z	7	832233	832371	145716	Mhap	/rhSeq-f/TAC ACC CNT TCG TTT AGC CrAT TTG /GT4/	/rhSeq-r/TGA TGT AAT CCC CTG CAC AGrC TCT G/GT4/
RH.C03EF5B621FF4C0Z0Z	7	1123202	1123330	151535	Mhap	/rhSeq-f/CGA GAT GTA AAC GAA GGT GArA AGG G/GT2/	/rhSeq-r/AGA CTC ACC AGA TTG ACC ArGA CTC /GT4/
RH.CBFE7DBF5A6E4D8Z0Z	7	1218911	1219036	153449	Mhap	/rhSeq-f/GAG ATT TTG CTG AAG TAC TAT AAG GrCA CGA /GT2/	/rhSeq-r/GAT AAT TTC CTT CAG CTC TGT CAA rGAC GT/GT2/
RH.0A6CDC396639498Z0Z	7	1427121	1427291	157614	Mhap	/rhSeq-f/GGT AGT CGC AAA GAA CAC TrCA NGT /GT1/	/rhSeq-r/CAG GAA ATT TGG AAA CGC CArG TAT G/GT3/
RH.82A3E1ACDE394D8Z0Z	8	168547	168665	165484	Mhap	/rhSeq-f/TGT TCC TCA CTT CTG AGA GTrA GAA G/GT3/	/rhSeq-r/GAC CAA GTG ACG AAG CArC TAC A/GT4/
RH.CC11B8DE53FA43DZ0Z	8	822406	822480	178560	Mhap	/rhSeq-f/AAA CCG AAC GTT TTA AAT GGG rCAC GT/GT2/	/rhSeq-r/GAC AGA ACC CAC TCG TAT ATC rCCA TT/GT2/
RH.4166D3F17C374C4Z0Z	8	1018073	1018248	182475	Mhap	/rhSeq-f/CCT CGA GAA GGC CAT AGT GrAG CAT /GT3/	/rhSeq-r/GAG AGG GTC ACC GGG TrCT AAG /GT3/
RH.6818F263362044BZ0Z	8	1157514	1157634	185264	Mhap	/rhSeq-f/AGA GGC TCC TAA AAG TGC TTrG TTA A/GT1/	/rhSeq-r/GTG GGT ACT CCT CAA GTG TTT rAAT AT/GT1/
RH.CD3AC424DC4D417Z0Z	8	1403792	1403883	190188	Mhap	/rhSeq-f/CAC GAA TAC ATG CAT GTG TGT rGCG CA/GT1/	/rhSeq-r/TCG TCG TCG TTA TGT ATG CTG rCAG TC/GT2/
RH.D5E3F1910FBA4C7Z0Z	8	1468975	1469148	191493	Mhap	/rhSeq-f/GAG ATC ACC AGA CCA CAG rGAG CA/GT2/	/rhSeq-r/CTG CAT TCA TGT CNT TCG AAA ArAT TGT /GT1/
RH.1B258A1478D74CDZ0Z	9	264626	264698	202627	Mhap	/rhSeq-f/GTT TGA GGA AAA TCT CGA AAG AAG AArC TAG C/GT3/	/rhSeq-r/TTG CTT ATC TCA GCA CTG CTT TrGG TCA /GT3/
RH.36E8980535C54B7Z0Z	9	448574	448630	206308	Mhap	/rhSeq-f/CCA GCT GTT TAT TTT CAA TCA AGT rGGT GA/GT1/	/rhSeq-r/CAC GAG AAA AGA AAA CGA AAT TGA rAAG CT/GT4/
RH.51F901C8B81F436Z0Z	9	756312	756407	212462	Mhap	/rhSeq-f/CAT GCC CAC GCA GGT ArCA CTC /GT4/	/rhSeq-r/CAC TCT CTN CAT GGG ATA ACT rAAA AC/GT2/

RH.C5069931FF214E6Z0Z	9	842507	842645	214187	Mhap	/rhSeq-f/TAA CCT CTT CAG CAT GAG AGT rCAT CG/GT4/	/rhSeq-r/CCA ATC GAA AGG TTG GCC ArCT TTA /GT4/
RH.290757F70A964E4Z0Z	9	1165616	1165689	220647	Mhap	/rhSeq-f/TTT GCC TCC CTA CTT GAA rGTA CG/GT2/	/rhSeq-r/CGA CAT TAA CCT GAA CAC CTrG GTC A/GT4/
RH.BD4EC483E885416Z0Z	9	1459600	1459729	226528	Mhap	/rhSeq-f/GTC CTG TTT TTG GAA AGG GTrA TNG T/GT3/	/rhSeq-r/CAA AGC TAG CTG CGT GGrG TTC T/GT2/
RH.52CD33BD1DC7471Z0Z	9	1688611	1688696	231108	Mhap	/rhSeq-f/AGC AAG GAC AAG ATG AGG ATrG AAC A/GT4/	/rhSeq-r/GCA TTT AAG GAC ATG CAA CTG rGAA TC/GT4/
RH.E03EC290DCDC420Z0Z	9	1864594	1864642	234628	Mhap	/rhSeq-f/CAT CAT CAC ATA TGC TAT CAT TGT rCTG CC/GT4/	/rhSeq-r/ACA AGT ACA AAA CGA TGA GCA AAA rGTG GA/GT2/
RH.E7EBCAE148AF44CZ0Z	9	2150871	2150956	240353	Mhap	/rhSeq-f/CTT CTG AAT TTT TCA TAA ATT CAT CCC rATT GC/GT1/	/rhSeq-r/GAG ATG GTT TAC CTT CAC TTC TrCA ACC /GT4/
RH.F6F8C3D0644D440Z0Z	10	203316	203481	246142	Mhap	/rhSeq-f/GCT CAC TTG GTT TCT TTT TAC CArG AAT G/GT2/	/rhSeq-r/AGG AAA GCA ACA GGG CAT rCCN AA/GT4/
RH.5B17C2D0F468424Z0Z	10	254364	254412	247163	Mhap	/rhSeq-f/GTT TTA CCA GAA TAT GTG GAG CAT rGAA AG/GT4/	/rhSeq-r/GCG TAT AGG ATC TGA ATA GTC ATC GrAT TAG /GT4/
RH.0D4A6DF10E97412Z0Z	10	479907	479908	MDR1_F1076L	Drug	/rhSeq-f/TTT AGG GAC ATC AAC TTC CCG rGCG TA/GT2/	/rhSeq-r/AGA CGC TAA TAA ATT CGA TGC TrCT GGG /GT2/
RH.45AE3D12F5854F9Z0Z	10	480206	480207	MDR1_Y976F	Drug	/rhSeq-f/TTC TTC TCT ACA TCC TTG TTG GrCT GCT /GT4/	/rhSeq-r/CTC ACT TTA TAG TGC TCT TCC TTrG TGA G/GT1/
RH.DEAB619F5DDE469Z0Z	10	725009	725169	256576	Mhap	/rhSeq-f/GCT GTT GAT ATC AAA TGT GCT rCGT CC/GT4/	/rhSeq-r/AAG AAG AGC AAG AAG GAG TTrC ACC C/GT4/
RH.C93D7255B094471Z0Z	10	848868	848916	259053	Mhap	/rhSeq-f/GTA AAA CTG TTT GAT ATC CCC GTT rGGT TA/GT4/	/rhSeq-r/CTT GGA AAG ACA ACA AGA AAC ArCG GAG /GT2/
RH.D97789F3C3B0468Z0Z	10	1097783	1097940	264031	Mhap	/rhSeq-f/GAT GAA TTC ATC CGT TTG GCrG ATG G/GT4/	/rhSeq-r/TGN AAA AGC TAA ACA TCC TAA ACrG AGC T/GT3/
RH.32854966B3C54A7Z0Z	10	1271498	1271644	267505	Mhap	/rhSeq-f/GCT AAT GTC TCT ACT AAC GTC TCT rACT AA/GT3/	/rhSeq-r/CAC GCA GGA GGC AAA NTA TrCA TTT /GT2/
RH.25183DF8EA034DAZ0Z	10	1408100	1408170	270238	Mhap	/rhSeq-f/GCA AAA TGA TGA GTA TTC CAT GAT TTT rCTG TG/GT1/	/rhSeq-r/GTA AAA AGG ATG CTC ATT TTG CTrG CAG G/GT1/
RH.6C64A045C9CA4D5Z0Z	11	75770	75798	274564	Mhap	/rhSeq-f/CCA ATT TAT GGT AGA GGA TTA GTA TCrA CTT G/GT3/	/rhSeq-r/CTT TAC TAA TTT CAG TTA TGT ATA ATG CCrC ATT G/GT1/
RH.3909CDB67E8543CZ0Z	11	177898	177971	276606	Mhap	/rhSeq-f/GGT TTT CAC TCC CTC CAC TrCA TTT /GT1/	/rhSeq-r/GGC ACT CTT TTG AGT AGC AGrC TTG A/GT3/
RH.E13F9A10B62A4BEZ0Z	11	607788	607927	285205	Mhap	/rhSeq-f/TAA CCA CCA CTG TGT TAT CCA TAT rCTG TT/GT1/	/rhSeq-r/TTA ATA AAG ACA CNA ATG TAG ATT TGA ACrA ACA T/GT3/
RH.83B95CBC9B044D2Z0Z	11	916515	916676	291380	Mhap	/rhSeq-f/GCA CTN TCT GAT AGC ATG TrGG TCT /GT4/	/rhSeq-r/GAG ACC CAT CCA CAT CTG rCGA AT/GT4/
RH.7D6C9D6F4F5044AZ0Z	11	1153666	1153850	296123	Mhap	/rhSeq-f/ATG TGA CGT CTC TCC ACC rCCC CT/GT2/	/rhSeq-r/CTA CAT CCA NCA TAC TCT GCrA GGT A/GT1/
RH.230B0FFB8743402Z0Z	11	1423369	1423519	301517	Mhap	/rhSeq-f/CAA AGT TGT AAA AGN GAT CTG CTC ArAA TTT /GT3/	/rhSeq-r/CAA GCC TGA CTG TTC AGA AAA ArAT TTC /GT1/

RH.9D2EBAFECC02434Z0Z	11	1572690	1572840	304503	Mhap	/rhSeq-f/GCC GCT CTA CAA GGG ArGA AGT /GT2/	/rhSeq-r/CCT TGC GCC TGA AGT TAT rCGT AC/GT1/
RH.2449A198108D4EDZ0Z	11	1742120	1742207	307891	Mhap	/rhSeq-f/GTT GTT AAC TCG TAA GCT GTT GArG GAA G/GT3/	/rhSeq-r/AGG CGA ATA ACC CAC GTA AGrG ACA A/GT3/
RH.DC722708FE7E46FZ0Z	11	2001515	2001699	313080	Mhap	/rhSeq-f/GCN CTT CAT GTT TAC AGT GTrA AGC A/GT3/	/rhSeq-r/TCA TTC GCC TCG ATG GAA rGAC AC/GT2/
RH.B289254C8C0F4D9Z0Z	12	324220	324242	322153	Mhap	/rhSeq-f/CTG GTG AAG GTG AAG GAA AAT GAA ArCT ATT /GT3/	/rhSeq-r/CCA TCT ATG TTT TCC GTT TTC TGC rGTC TT/GT3/
RH.B058277019D04D7Z0Z	12	532970	533101	326331	Mhap	/rhSeq-f/GAA ACG AAA TAT GCC GAC ATC TrAT TCT /GT1/	/rhSeq-r/TTG CAA AAA GCC GAA GGT TTT rCAC CA/GT2/
RH.64D26ADFAE5B4C9Z0Z	12	671183	671280	329094	Mhap	/rhSeq-f/AGT GGA ATT TGT AAA AAT ATT AAG TAT GArA GTA C/GT1/	/rhSeq-r/CTA TCA AAC ATG TCA ACG ACT GAA GrGA GAT /GT4/
RH.AB04234C54EB428Z0Z	12	995697	995758	335584	Mhap	/rhSeq-f/CAT ATG TGT TTG AAC GAT TCT TAC GTrA TGT T/GT1/	/rhSeq-r/TGG AAA AGC GAT TCA TAA TTT TTA GAG rCAA CG/GT1/
RH.F5FE56DD7B144F1Z0Z	12	1291327	1291407	341498	Mhap	/rhSeq-f/CTT TAT GTT GGA GAC TGA TTT GTT rCGC CT/GT1/	/rhSeq-r/GTG CTA ATC GAG AAG ATC CTA ACrG AAC G/GT3/
RH.D427FB9C888748FZ0Z	12	1457399	1457514	344819	Mhap	/rhSeq-f/GAG AAT AAA ATA CCC CTT CAA ATG GArG GAA G/GT1/	/rhSeq-r/CAT ATG TCA ATG TGA TTA CTT CTT GGT rGAA GC/GT4/
RH.E926E30943B14DCZ0Z	12	1880553	1880581	353283	Mhap	/rhSeq-f/GCT TGG TTT TCT GCA CCA GrGT CAT /GT3/	/rhSeq-r/TTG GAC GCC TTC CTG AAG rGAC TC/GT3/
RH.7B5B62F5A6C54CBZ0Z	12	1945902	1946055	354590	Mhap	/rhSeq-f/TGT ATN CTC CAT TTG GGA GTrC CAC C/GT1/	/rhSeq-r/CCA GAT GAG CAA CCC AArC GGA A/GT2/
RH.A4206DB085EE47DZ0Z	12	2108485	2108611	357841	Mhap	/rhSeq-f/TGA TGG TCT TTA CAC ACT CGT rATA GG/GT4/	/rhSeq-r/AAC CCG ATG ATG CCA TAG AArG GTG T/GT1/
RH.524A823035CE473Z0Z	12	2465991	2466085	364991	Mhap	/rhSeq-f/CCA TAT TTA TAT CTT CAT CAT CGC TTT TrCT TCC /GT3/	/rhSeq-r/ATT ATA AAT TCA GAC TCA TCT TAT TCA TCrC NAT G/GT4/
RH.8971DA4524DB42FZ0Z	12	2724027	2724063	370151	Mhap	/rhSeq-f/TCA CTC CTT TGC TCA GTC rCTG TT/GT4/	/rhSeq-r/GAC GAA ACG GAT AAA TTG CTA CArC TAC T/GT4/
RH.D7309B8EBCBD4ECZ0Z	13	167460	167596	382674	Mhap	/rhSeq-f/TGG ACG GCG ACA TCT GTrG AAC T/GT1/	/rhSeq-r/CGG AGC TGT TTA GCA GGT rCAT TC/GT2/
RH.F252700317D4403Z0Z	13	293615	293708	385196	Mhap	/rhSeq-f/AAC TGT CTC AGG TAA TTG CCrC CCT C/GT3/	/rhSeq-r/AGT TGG AAA GGA GAC AGA AAA ATrA TGG C/GT4/
RH.9E1CA1645B3C421Z0Z	13	485702	485833	389038	Mhap	/rhSeq-f/GTG GGA TGG TCT CTA CTT ATrG TGA C/GT3/	/rhSeq-r/CAA TAA CAG CTC CTT CAA CTT rCGA GA/GT4/
RH.CA5B246F7CB148AZ0Z	13	754813	754885	394419	Mhap	/rhSeq-f/GAA GTG TTA AAA TTA ACT GGA GCA ArAT ATG /GT4/	/rhSeq-r/GCA CAT CAT TGT AAT CCT GGA TrGA AGG /GT4/
RH.49DD6A84FF2848AZ0Z	13	1197713	1197831	403278	Mhap	/rhSeq-f/GCC GAC TAT CGC ACT TTT rGTT CT/GT2/	/rhSeq-r/TTG CAG AAG GAT GCT CTG ArAT GAG /GT4/
RH.28A3310975E14FDZ0Z	13	1865343	1865483	416631	Mhap	/rhSeq-f/TTT CCG TGG CTN AGT GGrC GAC T/GT1/	/rhSeq-r/AGG TGT CAG CGC TAG CrGG CAG /GT1/
RH.668CC46600DF42AZ0Z	13	1985698	1985814	419038	Mhap	/rhSeq-f/AAG AAA CTG CTA TAC TGT TTG CrCT ACG /GT4/	/rhSeq-r/ATC CAT TGA ATA ACC CGC TTT rGCA CT/GT3/

RH.AA0D417E764C4FDZ0Z	14	56852	57021	422331	Mhap	/rhSeq-f/CAA TAA ATC GAC CAA GNT TCT TTC CArG AAT A/GT4/	/rhSeq-r/TGC ATT TAT CTA TTA TGG TAG CAT AAT GGrA TAA T/GT4/
RH.EA14E24E533148FZ0Z	14	462743	462837	430447	Mhap	/rhSeq-f/CAG GCT CAT TGG AAT GGT TGrC TAC T/GT2/	/rhSeq-r/AAT ACA GAA GTG TAC CAA GCC rGTA GC/GT1/
RH.D19C9EEADCE7431Z0Z	14	675630	675699	434704	Mhap	/rhSeq-f/AAC GAC ATC CTC AAT TGG AAA rCAG GG/GT2/	/rhSeq-r/GTC CCA AAC TTT CAA GCT GTrA AAA G/GT1/
RH.FF5E58E56C4B4E5Z0Z	14	1206462	1206642	445323	Mhap	/rhSeq-f/CTT ATT GTG CAG GGA AAA CCA rCAA AT/GT4/	/rhSeq-r/TCA GGG AGC TAA ACG ATT ACA rGCA AC/GT1/
RH.2CD34BE4DF1F4B6Z0Z	14	1270400	1270401	DHPS_A553G	Drug	/rhSeq-f/CTG CAA CAG CTT AAT AGA CTG rGTC GT/GT2/	/rhSeq-r/AGC GTC GTT TTA ATG CAC AArG AGG G/GT2/
RH.C975D06C896447DZ0Z	14	1270910	1270911	DHPS_A383G	Drug	/rhSeq-f/AAC CTC ACA CTC CAA CTT ATG rCCA CT/GT3/	/rhSeq-r/CTT TTC AGA TGG CGG TTT ATT TrGT CGA /GT1/
RH.436418B818FD4C7Z0Z	14	1546237	1546249	452117	Mhap	/rhSeq-f/ATT GGG AAA CAG GAG AAA TGT TTA TrGG GTA /GT1/	/rhSeq-r/TAA GTC GTA ACC ATC AGG TAG TTT TrAT GAA /GT1/
RH.21577148368744AZ0Z	14	1763089	1763164	456454	Mhap	/rhSeq-f/CGC ATG CAA AAG GAA AAT TAA ATG TTrC ACT C/GT3/	/rhSeq-r/ACA AGA GTT ACA CTA TTC GCT TTT rGCG CT/GT3/
RH.04C2CDE0F619407Z0Z	14	1887637	1887677	458944	Mhap	/rhSeq-f/GTT AGA GAG TGG CAT GGA TGT rGAA TT/GT1/	/rhSeq-r/AAT ACC CGA GCA TCC TAA ACA rGAT CA/GT4/
RH.771A53FD18D3457Z0Z	14	2022558	2022663	461645	Mhap	/rhSeq-f/TCC CTT TTC TAT GAG GCT AAC TArG CTC T/GT3/	/rhSeq-r/GGC AAC GAA CTC ATC CAA TTA GrGA AAC /GT2/
RH.33061656A3E54DDZ0Z	14	2189680	2189767	464987	Mhap	/rhSeq-f/CCT GCA GTT TGC CTT TTT GrCA CAT /GT3/	/rhSeq-r/AGA ACC TCC ACG CAG TAC rCTG TT/GT1/
RH.0473747F9CDA4F7Z0Z	14	2261604	2261794	466426	Mhap	/rhSeq-f/ACT ATC TAA CGA GTA GCA GCrA GCG G/GT1/	/rhSeq-r/CGT TGT TNA GAG GTC CTC TrCT GGA /GT2/
RH.3AE56662E23E463Z0Z	14	2699925	2700045	475191	Mhap	/rhSeq-f/AAA ACG GTG CTC TTG TCG rGTG GT/GT4/	/rhSeq-r/GAA AAA GTG GGC CCG GTrG GAA A/GT2/
RH.BDE839E951D14CAZ0Z	14	2861965	2862134	478433	Mhap	/rhSeq-f/AAC TTA TTG AGG ATG TTA TGG AAG rAGN GA/GT4/	/rhSeq-r/CTT ACT GCA CCC AGA CAA TAA GrGA CTT /GT2/
RH.B3F556D4B68B487Z0Z	14	3009930	3010081	481392	Mhap	/rhSeq-f/GCG ATA TCA CTT TTT AAG TCA TCG rAAT GA/GT4/	/rhSeq-r/TAA GCC ACT AGN GTA TGA TGA CGrA ATT T/GT3/
RH.7E13E7FC3A8D47CZ0Z	MIT	2930	3141	MIT_Species2	Species	/rhSeq-f/CAT CGC AGC CTT GCA ATA AAT TAA TrAT TAT /GT1/	/rhSeq-r/CAG TCG AGT TCC TTT AAT GTA GTT TCrC TCA C/GT4/

*Mhap (microhaplotype), Drug (putative drug resistance marker), Species (mitochondrial *Plasmodium* spp. marker).

Supplementary Note 2. Bioinformatic Pipeline for rhAmpSeq data analysis

The snakemake-based pipeline provides two separate data analysis options that both require demultiplexed fastq files in a separate folder as input. As part of the pipeline, parameters are given in the config.yaml file, which can be modified as required.

a) The first analysis option performs SNP based variant calling using the provided reference genome PvP01_v2. The steps are as following:

(1) Generate manifest: This traverses the directory, identify the input fastq files and create a metadata file. This ensures that the forward and reverse reads belonging to the same sample are treated as such. The manifest is subsequently used by the pipeline to place the reads of each sample into its own directory adhering to the directory structure the pipeline requires.
(2) Mapping: The fastq files are mapped against the provided reference genome using bwa-mem2 and can be optionally trimmed prior. The alignment map (BAM) files are subsequently filtered to reads mapped in proper pairs.
(3) Base calibration: This step involves generating a recalibration table to detect systematic errors made by the sequencing machine when estimating the accuracy of each base call by comparing them to known sites of variation using GATK's BaseRecalibrator. The base quality scores in the input BAM file are then adjusted with GATK's ApplyBQSR according to the patterns identified in the recalibration table. The known variant database can be updated with knownvariants_dir in config file.
(4) Individual sample variant calling: Variant calling is performed for each sample against the reference genome using GATK's HaplotypeCaller with the following parameters "--max-reads-per-alignment-start 0 --do-not-run-physical-phasing --pileup-detection --dont-use-soft-clipped-bases". The parameters can be tweaked at the config file under haplotypecaller_flags.
(5) Joint variant calling: The GVCF outputs from previous step are used perform joint variant calling with GATK's GenotypeGVCFs. This step generates the final VCF outputs containing all the variants found in all the samples.

b) The second analysis option creates microhaplotypes from the same input files using 1) fasta files containing the forward and reverse primer sequences for all target markers, (2) a bed file containing the chromosome, start position, end position and name of each target marker, as well as (3) a fasta file of the target regions from the PvP01.v2 reference. The individual processes are listed here:

(1) Preparing the analysis directory: This step soft links the fastq files for each sample, making sure that it conforms to the requirements of subsequent steps of the pipeline.
(2) Trim: Trimming of adapters and primers using cutadapt with the options –pair-adapters which will pair every R1 with its corresponding R2 adapter and –discard-untrimmed which will remove read pairs with missing adapters and –action=trim to remove the adapter, it also have the option of performing poly-G trimming for sequencing with 2-dye chemistry.
(3) Mapping & filtering: The trimmed fastq files are mapped to the reference genome and any reads that cannot be mapped to the reference genome automatically discarded. The mapped reads are converted back into fastq files for the subsequent step.

(4) Run_dada2R: This process performs the dada2 based analysis of the trimmed files. The following parameters were used:
    (a) class: "parasite" as we looked at Plasmodium samples
    (b) maxEE: "5,5" this represents the maximum number of expected errors allowed
    (c) trim_right: "10,10": we noticed that the amplicon sequencing data occasionally lost quality in the last 4-10 reads requiring this option to cut off 10bp from the right
    (d) min_length: 30: discard reads that are shorter than 30bp
    (e) truncQ: "5,5" reads will be truncated as soon at the first base phred quality score is below 5
    (f) max_consist: 10 The maximum number of steps when selfConsist=TRUE
    (g) omegaA: 1e-120: treshold for significantly overabundance
    (h) justconcat: 0: turn off concatenation instead of merging
    (i) platform: "PE": set the platform to paired-end reads
    (j) trimqv: 15
(5) post_process: custom script that turns the dada2 output seqtab table into an ASV (Amplicon Sequence Variant) Table as well as and ASVSeqs fasta file using the (3) fasta file
(6) asv_to_cigar: this custom script transforms the ASVs into CIGAR (Concise Idiosyncratic Gapped Alignment Report) strings.
