# Supplementary material for: Microhaplotype deep sequencing assays to capture Plasmodium vivax infection lineages
Source: Nat Commun. 2025 Aug 5;16:7192. doi: 10.1038/s41467-025-62357-x (PMC12325997; doi:10.1038/s41467-025-62357-x)
Supplement: Supplementary file 3 — Description of Additional Supplementary Files [file 41467_2025_62357_MOESM3_ESM.pdf]

Description of Additional Supplementary Files

File Name: Supplementary Data 1
Description: Marker information.

File Name: Supplementary Data 2
Description: Sample information.

File Name: Supplementary Data 3
Description: Microhaplotype versus whole genome sequencing concordance.

File Name: Supplementary Data 4
Description: Within-host infection diversity.

File Name: Supplementary Data 5
Description: Randomized-controlled trial cases.

File Name: Supplementary Data 6
Description: Whole genome sequencing data information.
